# Maxillary Expansion and Its Effects on Circummaxillary Structures: A Review

**DOI:** 10.7759/cureus.33755

**Published:** 2023-01-13

**Authors:** Gauri V Patil, Pratiksha Lakhe, Priyanka Niranjane

**Affiliations:** 1 Dentistry, Sharad Pawar Dental College, Datta Meghe Institute of Higher Education and Research, Wardha, IND; 2 Orthodontics and Dentofacial Orthopedics, Sharad Pawar Dental College, Datta Meghe Institute of Higher Education and Research, Wardha, IND

**Keywords:** surgically assisted rapid maxillary expansion, slow maxillary expansion, rapid maxillary expansion, effects of maxillary expansion, maxillary expansion

## Abstract

Transverse maxillary discrepancies are the most common. The narrowed upper arch is the most prevalent problem an orthodontist encounter while treating adolescent and adult patients. Maxillary expansion is a technique used to increase the upper arch's transverse dimension to apply forces to widen the upper arch. For young children, a narrow maxillary arch has to be corrected using orthopedic and orthodontic treatments. In an orthodontic treatment plan, it is crucial to update transverse maxillary defeat. There are various clinical manifestations associated with a transverse maxillary deficiency which include a narrow palate, crossbite mainly seen in posteriors (unilateral or bilateral), severe crowding in anterior teeth, and cone-shaped hypertrophy can be seen. Some frequently used therapies for constricted upper arch include slow maxillary expansion, rapid maxillary expansion, and surgically assisted rapid maxillary expansion. Slow maxillary expansion requires light and constant force, whereas rapid maxillary expansion needs heavy pressure for activation. The surgical-assisted rapid maxillary expansion has gradually become popular to correct transverse maxillary hypoplasia. The maxillary expansion has various consequences on the nasomaxillary complex. There are multiple effects of maxillary expansion on the nasomaxillary complex. Mainly, the effect is seen on the mid-palatine suture along with the palate, maxilla, mandible, temporomandibular joint, soft tissue, and anterior and posterior upper teeth. It also affects functions like speech and hearing. Information on maxillary expansion is provided in depth in the following review article, along with its various effects on the surrounding structure.

## Introduction and background

Transverse maxillary deficiency is a common dentoskeletal complication. About 30% of patients receive any complicated orthodontic and surgical procedure it is because of transverse maxillary deficiency [1]. Maxillary transverse deficiency has been treated with maxillary expansions for years. In most cases, transverse maxillary discrepancies require orthodontic and orthopedic tooth movements.

1860 was the year in which Emerson Colon Angell introduced the concept of rapid maxillary expansion [2]. The work was not much appreciated during that time; however, presently, the procedure is widely used due to its simple and predictable result. The features seen clinically of transverse maxillary defects are characterized by posterior crossbite, which can be on one side or both sides, deep or narrow palatal vault, V-shaped palate, and crowding in anterior teeth [3-5]. Tooth size discrepancy between maxillary and mandibular is also standard [6]. In a situation with a complete crossbite, Liptook identified basic clinical features, including difficulty with nasal breathing, the volume of the nasal cavity reduced, breathing from the mouth, a narrow hard palate, and cone-shaped hypertrophy. The existence of at least two clinical features mentioned previously designates skeletal or dentoskeletal malocclusion and demands treatment which focuses on enlarging the transverse maxillary measurements [7].

Patients' age and malocclusion are considered when selecting treatment appliances [8,9]. By six years, normal palatal development has nearly been completed [10]. After puberty, the mid-palatal suture gradually becomes interdigitated, making separation difficult [11,12]. The posterior teeth are not able to move during the application of heavy or rapidly accelerating forces. Therefore, the forces are reflected in the mid-palatine suture. The suture opens up when the forces exceed the limit required for sutural resistance and tooth movement, while the teeth barely move. In addition to constriction of the periodontal ligament fibers, the alveolar process is bent by appliance, anchor teeth tipping, and the mid-palatine suture is opened gradually and other maxillary sutures.

Appliances used for rapid palatal expansion are tooth and tissue-borne and tooth-borne. The tooth-borne appliance contains only wires and bands with no acrylic covering. The appliances used are the Hyrax expander and the Issacson expander. The Hyrax is a single wireframe jackscrew without springs. Issacson appliance makes use of spring-loaded screws, which are joined on the first premolar and molar with bands [13]. Tooth and tissue-borne appliances include Hass and Derichsweiler applications for maxilla expansion. In slow maxillary expansion appliances, we used magnets, coffin springs, quad helix, NiTi expander, and many more.

Some of the immediate effect seen on the mid-palatine suture includes an expansion change of 1.52 to 4.3mm between the canine region mid-palatine suture, which represented 21.7% to 52.5% of a total expansion screw [14]. Mainly, changes in the expansion are seen in the molar region of the mid-palatine suture from 1.6 to 4.3mm, which accounts for 22.9% to 52.5% of the total expansion screw [14]. It is essential to inform the parent about the spacing between upper central incisors at the time of expansion. It is advocated that the expansion screw be rotated twice daily by the patients. This is associated with slight pain. Patients must be recalled every seven days, and many dentists recommend that the maxillary occlusal x-ray should be taken every seven days to see whether the mid-palatine suture is separate or not. It is important to stop activating the appliance right away if it is not effective, as it may result in alveolar fractures or damage to the periodontium. Treatment is required for two to three weeks, followed by three months of retention to ensure that the separate sutures can fill with bone [15]. This article will give a comprehensive review of types of maxillary expansion its advantages, disadvantages, indication and contraindications. Its effects on various structures of the Nasomaxillary complex.

## Review

**Figure 1:**
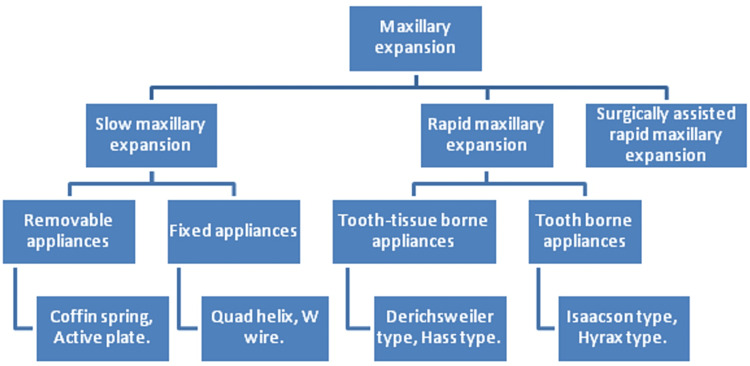
Maxillary expansion overview

When maxillary constriction is encountered, the treatment option is an expansion of the maxillary arch. Depending on the age and severity of constriction expansion can be slow, rapid and surgically assisted. A brief overview of maxillary expansion is given in Figure 1.

Types

Slow Maxillary Expansion

Slow palatal expansion is a technique used to correct the narrow upper arch by increasing it transversally. Slow palatal expansion, or dentoalveolar expansion, involves the use of appliances to widen the palate transversally. Even if the enlargement is only dental, skeletal alterations are still noticeable. There is less tissue resistance in structures around the maxilla, and there is an increase in bone formation in the mid-palatine suture. Therefore, it reduces the disadvantages of rapid maxillary expansion. Studies have shown that slow expansion promotes excellent post-expansion stability [16,17]. Force of 10-20 newtons must be enforced on the upper arch, which produces 450-900 g of force, which is not sufficient to separate a maturing suture [9,10,17-19]. Upper arch width ranges from 3.8 to 8.7mm with slow maxillary expansion by applying 900 gm of force for 1mm per week [18,20]. An appliance used for slow maxillary expansion is divided into two removable appliances and a fixed appliance. Active Plate, Shwartz Appliances, and Coffin Springs are examples of removable devices. Niti Palatal Expander, Quad Helix, and Spring Jet are examples of removable appliances.

The advantages of slow maxillary expansion are listed ahead. It exerts a constant physiological force. The front teeth are hardly or barely tipped. Anchored teeth experience a minimal amount of stress. The patient will find the device to be pleasant and light in weight [21]. Tendencies of recurrence are very few. Sutural integrity is maintained and is a little traumatic. Disadvantages include when compared to rapid maxillary expansion, a prolonged treatment period is necessary.

Slow maxillary expansion is indicated to correct minimal crowding by gaining spaces in patients with a crossbite. Slow palatal expansion is meant to exert slow continuous forces on patients who are having mild maxillary constriction with cleft lip and palate. While contraindicated in patients whose growth is completed.

Rapid Maxillary Expansion

As a routine clinical procedure, rapid expansion has become very common. The main aim of the rapid palatal expansion is to widen the narrow upper arch, but its impact is also seen on the bones of the head and face [22]. Within 2-4 weeks, an expansion (active) of 0.3 to 0.5mm occurs in the palate [23]. Cleft lip and palate birth abnormalities are among the most prevalent birth malformations, and the second is oral cleft [24,25]. As recently as the 1940s, Graber advocated Rapid Maxillary Expansion as a method to treat patients suffering from cleft lip and palate problems [26]. The main aim of the procedure is to separate the mid-palatine suture mechanically in the upper constricted arch, high palate, and complete crossbites. There is a decrease in overbite and an increase in overjet. Furthermore, it is used in situations of overcrowding by expanding the perimeter of the upper arch. The major obstacle to the expansion of palatal sutures is the sphenoid and zygomatic bone [13]. The appliance used for maxillary expansion is divided into tissue-borne and tooth-borne. Isaacson and Hyrax appliance is an example of tissue-borne appliances. While Derichsweiler and Hass appliances are examples of tooth-borne appliances.

The advantage of rapid maxillary expansion is short treatment time is required when compared to slow maxillary expansion. The disadvantages of rapid maxillary expansion are stated further. There is difficulty in maintaining oral cleanliness. It can also be removed or broken. There may be pain from the tissue infection (most common - acute ulcerative gingivitis). Sometimes sutures fail to open. Another drawback associated with the rapid maxillary expansion is that in post-retention assessment, relapse occurred mainly in the intercanine width of the upper arch. Rapid palatal expanders have several disadvantages, including discomfort caused by excessive forces, painful separation of the palatine suture, parental involvement in activating the appliance and bite opening [1].

Indicated in patients with unilateral or bilateral lateral discrepancies like posterior crossbite with multiple teeth [19]. Anterioposterior discrepancies also came under the indication of rapid maxillary expansion like malocclusions that include Class II division 1 and Class III. Patients with cleft lip and palate may also require rapid expansion. Single crossbite, open anterior bites, and a high mandibular plane angle are contraindications for rapid expansion. Uncooperative patients are also a contraindication for rapid expansion. Patients having severe vertical skeletal discrepancies along with asymmetrical upper or lower arches are contraindicated for rapid palatal expansion.

Surgically Assisted Rapid Maxillary Expansion

The surgical expansion has become one of the most widely accepted means to enlarge the upper arch in young patients. Orthodontic and surgical treatment align teeth and provide dental arch space. The strengthening impact in dentoskeletal expansion is provided by the zygomatic and sphenoid bones at the point of attachment to the maxillary arch. It establishes the integrity of the palatine suture and provides major resistance. Before undergoing surgery, appliances can be utilized to separate the roots of the upper incisors to prevent roots from being damaged by a midline maxillary surgical cut. Expansion generally occurs at a speech of 0.5mm each day, causing a gap between upper central incisors at midline diastema that must be informed to the patient/parents. It is the technique for patient without both sagittal and vertical deformities that may require upper arch surgery afterward [15,26].

Maxillary expansion in clinically mature individuals is accomplished with surgically assisted rapid palatal expansion which is its advantage [27]. The surgically assisted rapid palatal expansion has less risk of severe complications. The complications are epitasis, fracture of the base of the skull, reversible paralysis of the oculomotor nerve, and ophthalmic nerve discrepancies [28-30]. Post-operative bleeding, pain, and inflammation of the sinus are fewer significant challenges encountered: deviated nasal septum, expansion, which is not symmetrical, and relapse [31].

When extractions are contraindicated, surgically assisted rapid expansion is used to create space in a crowded maxillary arch. The goal of surgically assisted expansion is to widen maxillary hypoplasia caused by cleft palates. This is also used to narrow buccal corridors while smiling, correcting posterior teeth crossbite. Even if orthognathic surgery is anticipated, it is beneficial to broaden the maxilla first, as this will minimize the possibility of flaws and instability produced by segmental maxillary osteotomy. It is contraindicated in individuals with bleeding disorders to decrease the risk of severe bleeding [32,33].

Effects of maxillary expansion on surrounding structures

Effect on Mid-Palatine Suture

In a study done by Liu [14], effect of maxillary expansion on mid-palatine suture were studied. Immediate effects on mid-palatine suture are that in anterior palatal suture expansions is ranged from 2.42 to 4mm. From 0.84 to 2.88mm, the posterior mid-palatine suture shows expansion. Now let us see long-term effects on mid-palatine suture. Following rapid expansion therapy, the appliance is retained passively as long as three months and as long as 12 months. Mid-palatine suture opening did not change significantly with maxillary expansion, suggesting stable mid-palatine suture opening in the long term. In one study there was a triangular-shaped opening on the suture that was the largest at the anterior region [34].

Effect on Palatal Vault

A horizontal displacement of the maxillary halves resulted in a lowered palatine process in Haas study [35].

Effect on Maxilla

The periodontal ligament fibers are constricted, alveolar bone is bent, molars are tipped, and mid-palatal suture gradually opened. Mainly, the maxilla proceeds in forward and downward direction due to expansion [35].

Effects on Mandible

As the upper arch expansions, the mandible tends to swing downward and backward [13]. Mandibular plane angle is increases and open bite can be present.

Effects on Maxillary Anterior Teeth

There is spacing seen between central incisors is most spectacular effect associated with rapid maxillary expansion from patient’s perspective. According to an estimation, the incisors separate approximately half as much as an expansion screw has been opened during active suture opening [35].

Effects on Temporomandibular Joint

In a study by Arat et al., remodeling of the condylar branch was induced by rapid maxillary expansion. There is one report that found RME influenced condylar position and joint space [36], resulting in better uniformity between the side affected by crossbite and the side unaffected.

Effect on the Nasal Airway

Immediately following nasal expansion, the nasal cavity becomes wider, improving lung function. In the nasal cavity, the gain in width is on average 1.9mm but can exceed 8 to 10mm. The RME significantly increased the nasal cavity's interior dimensions, both in the front and back region of jaw and in the inferior and superior segments [37].

Effects on Speech

A study was done by Lubit, in which he investigated patients with deep palatal vault and at the end he concluded that patients with narrow arch have speech problem [38].

Effect on Hearing

In a study done by Braun, he stated that a narrow upper dental arch mainly causes nasal stenosis, and this can affect the Eustachian tube and middle ear and further results in hearing loss [39].

## Conclusions

Transverse maxillary discrepancies are widespread. The most common situation faced by an orthodontist while examining young patients is a narrow maxillary arch. Types of expansion include rapid expansion, slow expansion, and surgical expansion. In slow expansion, it will deliver a light and continuous force until the needed expansion is obtained, but a long treatment time is required. In rapid expansion, heavy force is exerted by the appliance. Treatment is shorter than slow expansion, but relapse can occur. The surgical expansion uses a combination of orthodontics and surgery to create a gap for the proper arrangement of teeth in the upper arch. There are various effects of maxillary expansion on surrounding structures, mainly mid-palatine suture, palate, maxilla, mandible, temporomandibular joint, and upper anterior and posterior teeth. Even effects on speech and hearing are seen.

## References

[1] Bailey LJ, White RP, Proffit WR, Turvey TA (1997). Segmental LeFort I osteotomy for management of transverse maxillary deficiency. J Oral Maxillofac Surg.

[2] Angle EH (1860). Treatment of irregularity of the permanent or adult teeth. Dental Cosmos.

[3] Ramieri GA, Spada MC, Austa M, Bianchi SD, Berrone S (2005). Transverse maxillary distraction with a bone-anchored appliance: dento-periodontal effects and clinical and radiological results. Int J Oral Maxillofac Surg.

[4] Pinto PX, Mommaerts MY, Wreakes G, Jacobs WV (2001). Immediate postexpansion changes following the use of the transpalatal distractor. J Oral Maxillofac Surg.

[5] Matteini C, Mommaerts MY (2001). Posterior transpalatal distraction with pterygoid disjunction: a short-term model study. Am J Orthod Dentofacial Orthop.

[6] Jadhav VV, Vasudevan SD, Kamble R, Tiwari MM (2020). Comparison of bolton’s ratio for evaluation of tooth size discrepancy between maxillary and mandibular arches in Vidarbha population. J Evolution Med. Dent. Sci.

[7] Laptook T (1981). Conductive hearing loss and rapid maxillary expansion. Report of a case. Am J Orthod.

[8] Ficarelli JP (1978). A brief review of maxillary expansion. J Pedod.

[9] Bell RA (1982). A review of maxillary expansion in relation to rate of expansion and patient's age. Am J Orthod.

[10] Moyers RE (1976). Standards of human occlusal development. https://babel.hathitrust.org/cgi/pt?id=mdp.39015007438222&view=2up&seq=7&ui=fullscreen.

[11] Persson M, Thilander B (1977). Palatal suture closure in man from 15 to 35 years of age. Am J Orthod.

[12] Handelman CS (1997). Nonsurgical rapid maxillary alveolar expansion in adults: a clinical evaluation. Angle Orthod.

[13] Bishara SE, Staley RN (1987). Maxillary expansion: clinical implications. Am J Orthod Dentofacial Orthop.

[14] Liu S, Xu T, Zou W (2015). Effects of rapid maxillary expansion on the midpalatal suture: a systematic review. Eur J Orthod.

[15] Gill D, Naini F, McNally M, Jones A (2004). The management of transverse maxillary deficiency. Dent Update.

[16] Cleall JF, Bayne DI, Posen JM, Subtelny JD (1965). Expansion of the midpalatal suture in the monkey. Angle Orthod.

[17] Storey E (1973). Tissue response to the movement of bones. Am J Orthod.

[18] Hicks EP (1978). Slow maxillary expansion: a clinical study of the skeletal versus dental response to low-magnitude force. Am J Orthod.

[19] Haas AJ (1965). The treatment of maxillary deficiency by opening the midpalatal suture. Angle Orthod.

[20] Rabah N, Al-Ibrahim HM, Hajeer MY, Ajaj MA (2022). Evaluation of rapid versus slow maxillary expansion in early adolescent patients with skeletal maxillary constriction using cone-beam computed tomography: a short-term follow-up randomized controlled trial. Dent Med Probl.

[21] Rabah N, Al-Ibrahim HM, Hajeer MY, Ajaj MA, Mahmoud G (2022). Assessment of patient-centered outcomes when treating maxillary constriction using a slow removable versus a rapid fixed expansion appliance in the adolescence period: a randomized controlled trial. Cureus.

[22] Ceylan I, Oktay H, Demirci M (1996). The effect of rapid maxillary expansion on conductive hearing loss. Angle Orthod.

[23] Graber TM, Swain BF (1975). Current orthodontic concepts and techniques. https://archive.org/details/cihm_98865/page/n11/mode/2up.

[24] Kamble RH, Shrivastav SS, Sangtani J, Ahuja MM, Bidwai P, Murarka S (2020). Assessment of change in SOC in parents participating in the treatment of their children having cleft lip & palate anomalies. J Evolution Med Dent Sci.

[25] Hazare A, Kamble R, Shrivastav S, Suroliya K, Hazare D, Bidwai P (2020). Association between genetic polymorphism in interferon regulatory factor 6 (IRF6) & non-syndromic cleft lip & palate cases in central Indian population. J Evolution Med Dent Sci.

[26] Susami T, Kuroda T, Amagasa T (1996). Orthodontic treatment of a cleft palate patient with surgically assisted rapid maxillary expansion. Cleft Palate Craniofac J.

[27] Al-Ouf K, Krenkel C, Hajeer MY, Sakka S (2010). Osteogenic uni- or bilateral form of the guided rapid maxillary expansion. J Craniomaxillofac Surg.

[28] Kraut RA (1984). Surgically assisted rapid maxillary expansion by opening the midpalatal suture. J Oral Maxillofac Surg.

[29] Messer EJ, Bollinger TE, Keller JJ (1979). Surgical-mechanical maxillary expansion. Quintessence Int Dent Dig.

[30] Pogrel MA, Kaban LB, Vargervik K, Baumrind S (1992). Surgically assisted rapid maxillary expansion in adults. Int J Adult Orthodon Orthognath Surg.

[31] Mehra P, Cottrell DA, Caiazzo A, Lincoln R (1999). Life-threatening, delayed epistaxis after surgically assisted rapid palatal expansion: a case report. J Oral Maxillofac Surg.

[32] Woods M, Wiesenfeld D, Probert T (1997). Surgically-assisted maxillary expansion. Aust Dent J.

[33] Koudstaal MJ, Poort LJ, van der Wal KG, Wolvius EB, Prahl-Andersen B, Schulten AJ (2005). Surgically assisted rapid maxillary expansion (SARME): a review of the literature. Int J Oral Maxillofac Surg.

[34] Weissheimer A, de Menezes LM, Mezomo M, Dias DM, de Lima EM, Rizzatto SM (2011). Immediate effects of rapid maxillary expansion with Haas-type and hyrax-type expanders: a randomized clinical trial. Am J Orthod Dentofacial Orthop.

[35] Haas AJ (1961). Rapid expansion of the maxillary dental arch and nasal cavity by opening the midpalatal suture. Angle Orthod.

[36] Leonardi R, Caltabiano M, Cavallini C, Sicurezza E, Barbato E, Spampinato C, Giordano D (2012). Condyle fossa relationship associated with functional posterior crossbite, before and after rapid maxillary expansion. Angle Orthod.

[37] Gray LP (1975). Results of 310 cases of rapid maxillary expansion selected for medical reasons. J Laryngol Otol.

[38] Lubit EC (1967). The relationship of malocclusion and faulty speech articulation. J Oral Med.

[39] Braun F (1966). A contribution to the problem of bronchial asthma and extension of the palatine suture. Rep Congr Eur Orthod Soc.

